# Higher serum ferritins are associated with higher blood pressure: A cross-sectional study

**DOI:** 10.1097/MD.0000000000037485

**Published:** 2024-03-22

**Authors:** Shaoli Li, Feilong Chen, Tao Li, Yijing Cheng, Guimin Huang, Dongqing Hou, Wenqian Liu, Tao Xu, Junting Liu

**Affiliations:** a Child Health Big Data Research Center, Capital Institute of Pediatrics, Beijing, China; b Department of Epidemiology and Statistics, Institute of Basic Medical Sciences, Chinese Academy of Medical Sciences and School of Basic Medicine, Peking Union Medical College, Beijing, China.

**Keywords:** adults, hypertension, NHANES, serum ferritin, United States

## Abstract

The aim of the study was to investigate the association between serum ferritin and hypertension among American adults from National Health and Nutrition Examination Survey (NHANES) 1999 to 2018. A total of 16,125 participants were included. Weighted logistic regression and subgroup analyses were performed to explore the association. We found that serum ferritin was closely correlated to hypertension. Individuals with high serum ferritin were more likely to have higher systolic or diastolic blood pressure (SBP, DBP) than those with lower serum ferritin. Restricted cubic spline showed a significant non-linear association between serum ferritin and SBP/DBP. Higher level of serum ferritin (Q3 74.1–147 μg/L and Q4 > 147 μg/L) was found to have positive association with high SBP [Q3 (OR: 1.246, 95% CI:1.020–1.523), Q4 (OR: 1.354, 95% CI:1.096–1.674)], and hypertension [Q3 (OR: 1.283, 95% CI:1.099–1.499), Q4 (OR: 1.424, 95% CI:1.197–1.63)] in the whole population. In people aged between 20 and 60, subjects with high serum ferritin were significantly associated with a higher risk of hypertension, but in those over 60, the relationship between serum ferritin level and hypertension is negative. A non-linear association between serum ferritin and SBP, as well as DBP, was discovered. There was age difference in association between serum ferritin and hypertension in American adults, and further researches were needed to understand the mechanisms behind the difference.

## 1. Introduction

Hypertension is a very important controllable risk factor for the morbidity and mortality of cardiovascular and cerebrovascular diseases (CVD) in the world, and is also the most common chronic disease in clinical practice. The occurrence of hypertension is closely related to diet, environment, genetics, lifestyle, and other factors.^[1–5]^ Its main pathophysiology focuses on: Increased vascular inflammation, vascular remodeling, increased vascular contractility and oxidative stress, etc., control of hypertension and prevention of complications have become major world public problems. It has been documented that hypertension is the most dangerous factor for heart disease and stroke, which are two leading causes of death for Americans.^[6]^ In the United States, nearly half of adults (47.3% or 116.0 million) live with hypertension.^[7,8]^ A total of 4,270,415 hypertension – related CVD deaths were reported during 2000 to 2019 among US adults aged ≥ 35 years, representing 8.8% of all deaths and 25.4% of CVD deaths during this period.^[9]^ This burden costs about 48.6 billion dollars annually, including spending on health care services and antihypertensive medications, and on productivity loss from premature death.^[10]^ Thus, there is an urgent need to prevent and control hypertension to reduce the morbidity and mortality of hypertension related diseases, and reduce the burden.

Ferritin is a ubiquitous protein involved in the regulation of iron homeostasis, and serum ferritin is often used to reflect the iron storage status, and involved in a wide range of physiological and pathological processes. Serum ferritin concentrations varied across different sociodemographic, lifestyle, and dietary factors in Chinese population. A higher intake of red meat was associated with higher ferritin concentrations in men and older women.^[11]^ Food fortification with multiple micronutrients may improve serum ferritin.^[12,13]^ Iron fortification led to a significant increase in serum ferritin and hemoglobin levels in women of reproductive age and pregnant women.^[14]^

Serum ferritin could reflect systemic inflammatory status and oxidative stress-mediated cellular damage.^[15–17]^ Hypertensive patients, often characterized by chronic inflammation and oxidative stress damage.^[18–20]^ Thus, serum ferritin may affect the risk of hypertension through inflammation and oxidative stress.

Therefore, in this study, we investigated data from National Health and Nutritional Examination Survey (NHANES) 1999–2018 and investigated the correlation between serum ferritin and hypertension status in American adult population. Subgroup analysis was conducted to further explore the associations in various stratified groups.

## 2. Materials and methods

### 2.1. Participants

Data were obtained from the NHANES, a series of cross-sectional surveys which aimed to investigate the nutrition and health status of the US population.^[21]^ The survey used a complex multi-stage sampling design to obtain a representative, non-institutionalized sample of the US population.^[22]^ Detailed sampling designs were available elsewhere. The study was proved by the Ethics Committee^[23]^ and all subjects had signed informed consent forms.

**Figure 1.**
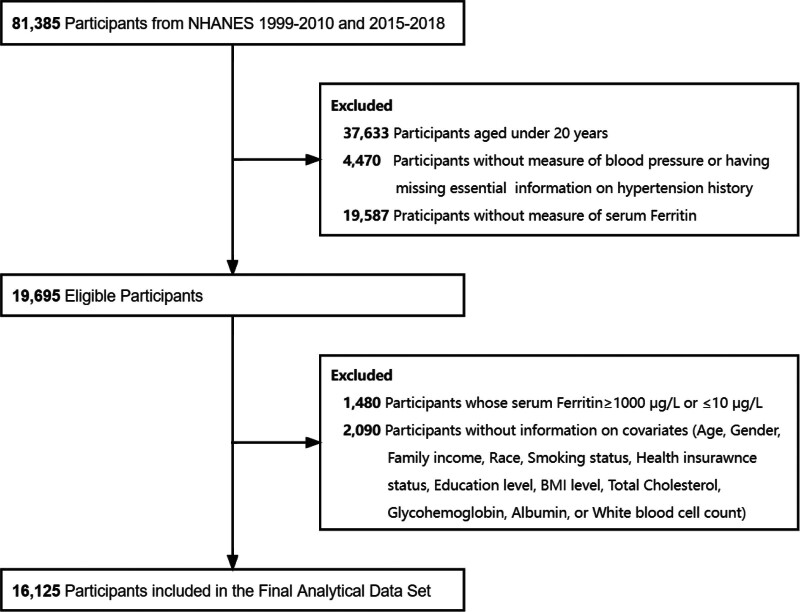
Flowchart of participants selection.

In this study, subjects from 8 survey cycles, 1999 to 2010 and 2015 to 2018, were included. Those who under 20 years of age (n = 37,633), lack of serum ferritin measurements (n = 19,587), missing information on blood pressure measurements and essential details on hypertension history (n = 4470), abnormal serum ferritin value that greater than 1000 µg/L or less than 10 µg/L (n = 1480) and missing information on covariates (n = 2090) were excluded from this study. After excluding the individuals based on the above-mentioned criteria, 16,125 participants (5464 males and 10,661 females) were included (Fig. 1).

### 2.2. Blood pressure

Blood pressure was measured in standardized mobile medical examination centers (MCEs) and were taken by one of the medical examination centers examiners, who had received prior training and got licensed before they were allowed to perform. Three consecutive blood pressure readings were obtained after participants were asked to rest quietly in a sitting position for 5 minutes.^[24]^ The average of the 3 systolic blood pressure (SBP) readings was taken as the final SBP record and the 3 diastolic blood pressure (DBP) mean values was taken as the final DBP record.

In addition, subjects were asked if they have ever been diagnosed with hypertension and if they had taken antihypertensive medications during the in-home interview. Respondents with any of the following characteristics were classified as hypertensive: SBP ≥ 140 mm Hg; DBP ≥ 90 mm Hg; self-reported doctor-diagnosed high blood pressure; or the use of antihypertensive medications.

### 2.3. Measurement of serum ferritin

Blood samples were collected at the medical examination centers and transported centrally to the third-party laboratories for testing. The method principle for measurement of ferritin was immuno-turbidimetry. The serum ferritin was divided into 4 groups based on quartiles, Q1 (ferritin ≤ 36 μg/L), Q2 (ferritin 36.1–74 μg/L), Q3 (ferritin 74.1–147 μg/L), and Q4 (ferritin > 147 μg/L).

### 2.4. Covariates

The following factors were used as covariates: Age, gender, race, education level, family income, smoking status, physical activity, body mass index (BMI) and health insurance status. Additionally, we adjusted for hematological indicators including total cholesterol, glycohemoglobin, albumin and white blood cell count. Data on demographic and lifestyle characteristics were obtained through in-home interviews, physical examination indicators were measured at the MCEs, and hematological indicators were tested by the unified third-party laboratories.

Age was divided into 3 groups, the youth (aged between 20 and 40), the middle-aged (aged between 41 and 60) and the elders (aged over 60). Race was divided into 4 groups, non-Hispanic white, non-Hispanic black, Hispanic and others. Family income status was assessed using the family poverty-to-income ratio (family PIR), which was calculated by dividing family income by the poverty threshold for the survey year.^[25]^ The PIR was used to define 2 categories of income status: low (PIR ≤ 1.3), and high (PIR > 1.3). Educational level was classified as pre-high school, high school graduate or general educational development, and college or above. Subjects were defined as “covered by health insurance” if they had health insurance or other types of health care coverage. When asked “Did you smoke at least 100 cigarettes in life,” a negative answer was defined as “nonsmoker.” If the answer is “Yes,” but the answer to “Do you now still smoke?” was “No,” then they were classified as “Former smoker.” A “Yes” answer was defined as “Current smoker.” Participants were classed as inactive if they reported fewer than 10 min of moderate-to-vigorous physical activity per week.^[26]^ BMI was calculated as body mass (kg)/height (m) squared. Total cholesterol (mg/dL), albumin (mg/dL), total white blood cell count (SI), and glycohemoglobin (%), were also included as continuous covariate.

### 2.5. Definition of specific disease population

Subjects were defined as “Abnormal liver function” if their AST > 80 U/L or ALT > 80 U/L^[27]^ or if he/she answered “yes” to “Ever told you had any liver condition?.” The subject’s estimated glomerular filtration rate was calculated using the CKD-Epi formula.^[28]^ Kidney disease was defined if their estimated glomerular filtration rate were < 60 mL/min/1.73 m^2^ or if the they had been told that they had any kidney condition. Subjects with any following characteristics were classified as dyslipidemia: LDL ≥ 194 mg/dL; triglycerides greater than 885 mg/dL; and HDL < 25 mg/dL.^[29]^

### 2.6. Statistical analysis

Sampling weights were calculated following the NCHS Analytic Guidelines,^[30]^ and all analysis were weighted unless otherwise specified. For continuous variables, data were expressed as mean ± standard deviations, the differences among ferritin groups were tested using weighted linear regression models. For categorical variables, data were described by frequencies and percentages, differences among groups were compared using weighted Rao-Scott χ^2^ tests. Simultaneously, *P* trend analysis was performed in the weighted regression analyses by considering the quartiles as a continuous variable.

We utilized the lowest serum ferritin level as reference. Weighted logistic regression analysis were performed to estimate the strength of the association between high SBP (≥140 mm Hg), high DBP (≥90 mm Hg), hypertension and serum ferritin levels in the whole sample population. Odds ratios (ORs) and their 95% confidence intervals (CIs) were calculated to evaluate the association. Three independent models were conducted. Model 1 did not adjust any confounders. Model 2 adjusted age, gender, race, education level, family income, smoking status, physical activity, BMI and health insurance status. Model 3 further adjusted blood indicators and specific diseases including total cholesterol, glycohemoglobin, albumin, white blood cell count, liver disease, kidney disease and dyslipidemia. Model 3 was first performed on the whole sample and then stratified by age and gender. Age was not adjusted when analyzing was conducted in age-stratified models, and similarly gender was not adjusted in gender-stratified model. Likelihood ratio tests were used to test the interaction of stratification factors. Linear trends between ferritin quartiles were tested using weighted linear regression analysis. To clearly illustrated the association between serum ferritin and hypertension, we modeled ferritin against hypertension and used a restricted cubic spline (RCS) with 5 knots located at the 5th, 27.5th, 50th, 72.5th, and 95th percentiles to flexibly model the underlying relationship.

Subgroup analyses were performed to further assess the robustness of the association between serum ferritin and hypertension in subjects with specific diseases. In people with abnormal liver function, kidney diseases and dyslipidemia, we adjusted confounders based on 3 independent models mentioned above to explore the association, and potential interactions were tested as well.

All analyses were performed using SAS 9.4 (SAS Institute, Cary, NC) and R 4.2.2, and a *P* value < .05 was considered statistically significant.

## 3. Results

### 3.1. Characteristics of participants

A total of 16,125 participants (5464 males and 10,661 females) were included in our study. The prevalence of hypertension of whole population was 34.66% (5589/16,125), with a significantly higher prevalence in males (42.9%, 2346/5464) than in females (30.4%, 3243/10,661). Males had significantly higher levels of serum ferritin than females (*P* < .001). The baseline characteristics of the study participants according to serum ferritin levels were shown in Table 1. The proportion of subjects with elevated serum ferritin increases with age (*P* for trend < .001). In comparison to those who were normal weight or underweight, the percentage of people with higher serum ferritin levels was substantially greater in the overweight or obese subjects. With rising serum ferritin levels, SBP, DBP, and hypertension prevalence all significantly increased (all *P* for trend < .001). Total cholesterol, HDL, LDL, triglycerides, glycated hemoglobin and albumin also showed an ascending trend with increasing serum ferritin levels.

**Table 1:** Characteristics of participants aged over 20 years by serum ferritin levels from NHANES 1999–2010 and 2015–2018.

Variables	Q1 ≤36 μg/L	Q2 36.1–74 μg/L	Q3 74.1–147 μg/L	Q4 >147 μg/L	*F*/χ^2^*	*P* value†	*P* for trend‡
Sample size	n = 4096	n = 4035	n = 3976	n = 4018			
Serum ferritin, μg/L	23.16 ± 7.50	54.10 ± 11.08	105.88 ± 20.72	282.14 ± 145.07	34,538.6	<.001	<.001
SBP, mm Hg	116.0 ± 17.0	119.7 ± 19.2	125.1 ± 20.5	128.5 ± 19.2	51,909.2	<.001	<.001
DBP, mm Hg	68.4 ± 11.5	69.7 ± 11.7	71.9 ± 12.5	73.9 ± 12.4	29,676.3	<.001	<.001
Total cholesterol, mg/dL	192.77 ± 41.20	192.91 ± 38.67	197.53 ± 41.96	201.57 ± 44.98	25,438.8	<.001	<.001
HDL-cholesterol, mg/dL	56.14 ± 15.89	53.12 ± 15.40	50.33 ± 14.98	47.84 ± 14.97	6508.87	<.001	<.001
LDL-cholesterol, mg/dL	110.24 ± 35.12	112.98 ± 34.15	117.82 ± 36.85	121.27 ± 36.47	8294.73	<.001	<.001
Triglyceride, mg/dL	118.70 ± 83.98	117.39 ± 99.19	130.85 ± 94.33	158.24 ± 178.26	1613.72	<.001	<.001
Glycohemoglobin, %	5.35 ± 0.73	5.47 ± 0.85	5.62 ± 0.98	5.86 ± 1.31	69,068.7	<.001	<.001
Albumin, g/dL	4.07 ± 0.42	4.19 ± 0.36	4.25 ± 0.36	4.27 ± 0.36	99,959.9	<.001	<.001
White Blood Cell Count, SI	7.60 ± 2.25	7.43 ± 2.24	7.34 ± 2.43	7.38 ± 6.58	13,442.8	<.001	.671
Hypertension							
No	3191 (77.91)	2871 (71.15)	2371 (59.63)	2103 (52.34)	103.25	<.001	<.001
Yes	905 (22.09)	1164 (28.85)	1605 (40.37)	1915 (47.66)			
SBP level							
<140 mm Hg	3745 (91.43)	3504 (86.84)	3200 (80.48)	3087 (76.83)	50.25	<.001	<.001
≥140 mm Hg	351 (8.57)	531 (13.16)	776 (19.52)	931 (23.17)			
DBP level							
<90 mm Hg	3957 (96.61)	3874 (96.01)	3711 (93.34)	3668 (91.29)	28.73	<.001	<.001
≥90 mm Hg	139 (3.39)	161 (3.99)	265 (6.66)	350 (8.71)			
Age, yr							
20–40	2557 (62.43)	2073 (51.38)	1492 (37.53)	1034 (25.73)	72.36	<.001	<.001
41–60	1129 (27.56)	1151 (28.53)	1308 (32.90)	1453 (36.16)			
≥60	410 (10.01)	811 (20.10)	1176 (29.58)	1531 (38.10)			
Gender							
Male	284 (6.93)	782 (19.38)	1679 (42.23)	2719 (67.67)	653.53	<.001	<.001
Female	3812 (93.07)	3253 (80.62)	2297 (57.77)	1299 (32.33)			
Ethnicity							
Non-Hispanic white	1818 (44.38)	1932 (47.88)	1907 (47.96)	1779 (44.28)	4.74	<.001	.2744
Non-Hispanic black	749 (18.29)	659 (16.33)	757 (19.04)	888 (22.10)			
Hispanic	1247 (30.44)	1118 (27.71)	988 (24.85)	945 (23.52)			
Other	282 (6.88)	326 (8.08)	324 (8.15)	406 (10.10)			
Family income to poverty ratio (PIR)							
≤1.30	1315 (32.10)	1160 (28.75)	1118 (28.12)	1062 (26.43)	7.60	<.001	<.001
>1.30	2781 (67.90)	2875 (71.25)	2858 (71.88)	2956 (73.57)			
Education							
Less than High School	1850 (45.17)	1794 (44.46)	1903 (47.86)	2121 (52.79)	7.93	<.001	<.001
High School Graduates or GED	1285 (31.37)	1272 (31.52)	1180 (29.68)	1108 (27.58)			
Some College or above	961 (23.46)	969 (24.01)	893 (22.46)	789 (19.64)			
Body mass index							
Underweight	150 (3.66)	151 (3.74)	126 (3.17)	119 (2.96)	25.00	<.001	<.001
Normal weight	1296 (31.64)	1340 (33.21)	1111 (27.94)	908 (22.60)			
Overweight or obesity	2650 (64.70)	2544 (63.05)	2739 (68.89)	2991 (74.44)			
Smoking status							
Nonsmoker	2697 (65.84)	2402 (59.53)	2089 (52.54)	1957 (48.71)	24.89	<.001	<.001
Former smoker	643 (15.70)	790 (19.58)	1000 (25.15)	1172 (29.17)			
Current smoker	756 (18.46)	843 (20.89)	887 (22.31)	889 (22.13)			
Covered by health insurance							
No	873 (21.31)	805 (19.95)	759 (19.09)	708 (17.62)	0.47	.701	.3441
Yes	3223 (78.69)	3230 (80.05)	3217 (80.91)	3310 (82.38)			
Pregnant history (only female)							
No	913 (23.95)	806 (24.78)	476 (20.72)	208 (16.01)	16.40	<.001	<.001
Yes	2899 (76.05)	2447 (75.22)	1821 (79.28)	1091 (83.99)			
Suffering from abnormal liver function formerly or presently							
No	3998 (97.61)	3884 (96.26)	3781 (95.10)	3669 (91.31)	36.64	<.001	<.001
Yes	98 (2.39)	151 (3.74)	195 (4.90)	349 (8.69)			
Suffering from kidney disease formerly or presently							
No	3014 (73.58)	2925 (72.49)	2734 (68.76)	2653 (66.03)	11.31	<.001	<.001
Yes	1082 (26.42)	1110 (27.51)	1242 (31.24)	1365 (33.97)			
Suffering from dyslipidemia formerly or presently							
No	1375 (33.57)	1509 (37.40)	1819 (45.75)	2140 (53.26)	53.27	<.001	<.001
Yes	2721 (66.43)	2526 (62.60)	2157 (54.25)	1878 (46.74)			

Data are presented as means ± standard deviation for continuous variables, and frequencies and percentage in colon (%) in parenthesis for categorized variables.

BMI = body mass index, DBP = diastolic blood pressure, GED = general education development, HDL-cholesterol = high-density lipoprotein cholesterol, LDL-cholesterol = low-density lipoprotein cholesterol, SBP = systolic blood pressure.

* Differences between groups were compared on the basis of *F* value for continuous variables and *χ*^2^ value for categorized variables.

† *P* value for characteristics comparison among serum ferritin levels estimates based on weighted linear regression model for continuous variables and Rao-Scott chi-square tests for categorical variables.

‡ *P* for trend is used to test linear trends among different serum ferritin levels based on weighted linear regression model.

### 3.2. Distribution of serum ferritin

Table 2 displays the geometric means and percentiles for serum ferritin. The geometric mean and standard error of serum ferritin were 96.38 ± 1.85 in subjects with hypertension, significantly higher than those without hypertension (66.71 ± 0.86). Similarly, individuals with high SBP or high DBP were more likely to have higher serum ferritin than those with normal SBP and DBP values.

**Table 2:** Distribution of serum ferritin (μg/L).

		GM ± Se	Min	10th	25th	50th	75th	90th	Max
Overall		74.55 ± 0.91	10.1	20.0	36.0	74.0	147.0	261.0	997.0
Hypertension	Hypertension	96.38 ± 1.85	10.1	26.7	52.0	103.0	188.0	337.0	995.0
	Non-Hypertension	66.71 ± 0.86	10.1	18.7	31.0	62.0	124.0	222.0	997.0
SBP level	High SBP (≥140 mm Hg)	100.88 ± 2.70	10.2	30.5	57.0	111.0	196.0	350.0	995.0
	Normal SBP (<140 mm Hg)	71.49 ± 0.88	10.1	19.0	33.9	68.0	137.0	244.0	997.0
DBP level	High DBP (≥90 mm Hg)	108.97 ± 5.12	10.9	28.2	59.3	118.0	207.0	351.0	966.0
	Normal DBP (<90 mm Hg)	72.97 ± 0.88	10.1	20.0	35.0	72.0	143.0	254.0	997.0

GM = geometric mean, Se = standard error.

### 3.3. Association between serum ferritin and blood pressure

**Figure 2.**
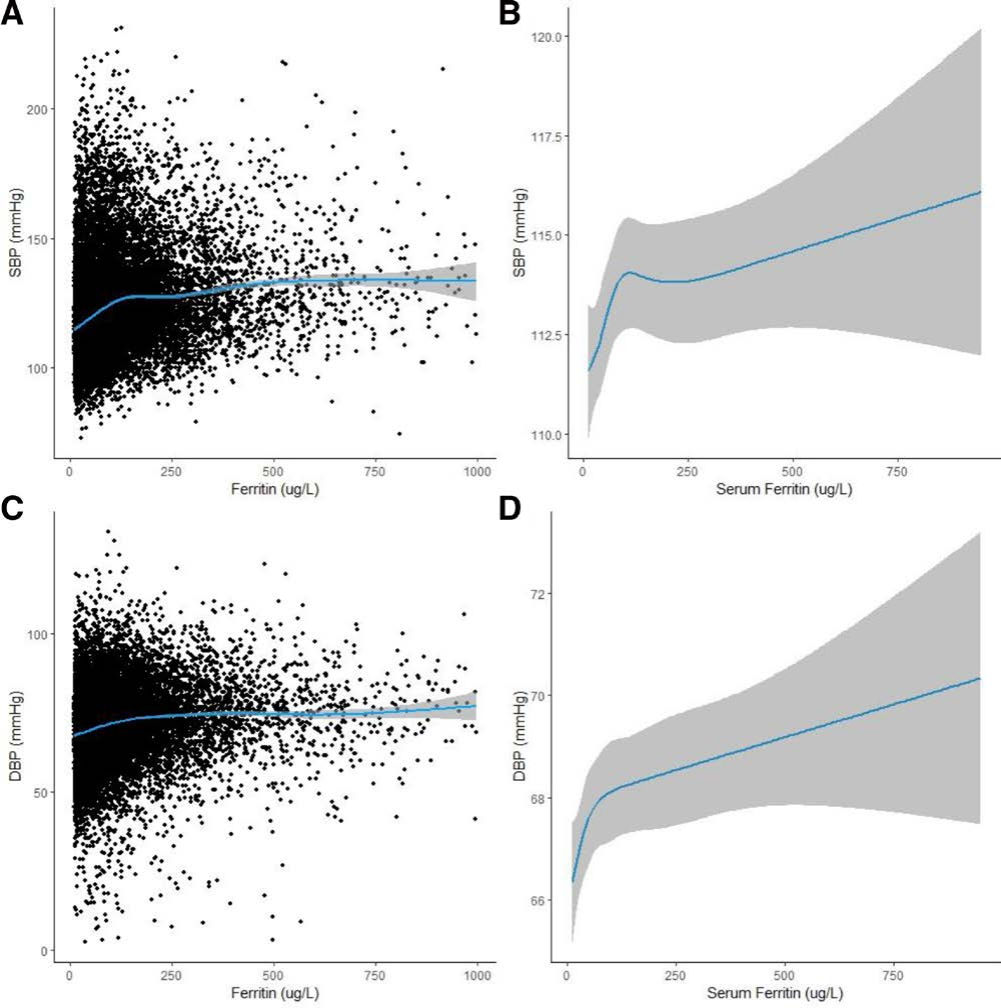
Association between serum ferritin with SBP/DBP; A: scatter plots of ferritin and SBP, each black point represents a sample, blue line represents smooth curve fit between variables, and shaded areas represent 95% CI from the fit. B: serum ferritin restricted cubic spline regression with 5 knots in all participants; shaded areas inside the dashed lines are 95% CIs. C: scatter plots of ferritin and DBP, each black point represents a sample, blue line represents smooth curve fit between variables, and shaded areas represent 95% CI from the fit. D: serum ferritin restricted cubic spline regression with 5 knots in all participants; shaded areas inside the dashed lines are 95% CIs. Two RCS regression models adjusted covariate including age, gender, race, family income, education level, BMI, smoking status, physical activity, health insurance status, total cholesterol, glycohemoglobin, albumin, white blood cell count, liver disease, kidney disease and dyslipidemia. CI = confidence intervals, DBP = diastolic blood pressure, RCS = restricted cubic spline, SBP = systolic blood pressure.

We plotted scatter plots and fitted curves for serum ferritin with SBP and DBP, respectively. The fitted curves showed a non-linear trend at certain concentrations of serum ferritin (Fig. 2A and C). To further explore the non-linear relationship between serum ferritin and SBP/DBP, RCS models were fitted with 5 nodes of 5th, 27.5th, 50th, 72.5th, and 95th percentiles. RCS showed a significant non-linear association between serum ferritin and both SBP and DBP (Fig. 2B: *P* for nonlinear = .015, Fig. 2D: *P* for nonlinear = .038). The influence was much increased to the left of the inflection point whereas it had little effect to the right.

Weighed logistic regression models were used to assess the association between serum ferritin and high SBP, high DBP, and hypertension in the entire population. High SBP, high DBP, and hypertension were all significantly associated with serum ferritin levels (Model 1, Table 3). Serum ferritin levels remained to be significantly associated with high SBP and hypertension after adjusting for covariates, but not with high DBP. Compared to the Q1 serum ferritin level, the risk of hypertension was 1.283 times higher in Q3 (95% CI: 1.099–1.499) and 1.424 times higher in Q4 (95% CI: 1.197–1.693). As serum ferritin levels elevated, the risk of hypertension also increased, with a statistically significant trend (*P* for trend < .0001). Serum ferritin was significantly associated with high SBP, high DBP, and hypertension when it was included as a continuous variable in the model with or without adjusting for confounders. Each 10 μg/L increase in serum ferritin was positively associated with a 0.8% increase in the risk of high SBP (95% CI: 1.002–1.014), 0.8% increase in the risk of high DBP (95% CI: 1.002–1.017) and 1.2% increase in the risk of hypertension (95% CI: 1.007–1.016) in Model 3.

**Table 3:** Association between serum ferritin levels and risk of high SBP (≥140), high DBP (≥90), and hypertension among American adults: NHANES 1999–2010 and 2015–2018.

	Case	Model 1	Model 2	Model 3
High SBP (≥140 mm Hg)				
Serum ferritin/(10 μg/L)		1.025 (1.020, 1.029)*	1.009 (1.003, 1.015)*	1.008 (1.002, 1.014)*
Q1 (≤36 μg/L)	351 (8.57)	1 (ref)	1 (ref)	1 (ref)
Q2 (36.1–74 μg/L)	531 (13.16)	1.358 (1.146, 1.610)*	1.085 (0.896, 1.314)	1.077 (0.890, 1.303)
Q3 (74.1–147 μg/L)	776 (19.52)	2.026 (1.704, 2.409)*	1.262 (1.028, 1.549)*	1.246 (1.020, 1.523)*
Q4 (>147 μg/L)	931 (23.17)	2.646 (2.228, 3.143)*	1.413 (1.140, 1.751)*	1.354 (1.096, 1.674)*
* P* for trend		<.0001	.0012	.0084
High DBP (≥90 mm Hg)				
Serum ferritin/(10 μg/L)		1.027 (1.021, 1.034)*	1.012 (1.003, 1.020)*	1.009 (1.002, 1.017)*
Q1 (≤36 μg/L)	139 (3.39)	1 (ref)	1 (ref)	1 (ref)
Q2 (36.1–74 μg/L)	161 (3.99)	0.957 (0.693, 1.323)	0.883 (0.629, 1.239)	0.862 (0.616, 1.207)
Q3 (74.1–147 μg/L)	265 (6.66)	1.734 (1.314, 2.287)*	1.243 (0.903, 1.712)	1.182 (0.861, 1.623)
Q4 (>147 μg/L)	350 (8.71)	2.569 (1.964, 3.359)*	1.337 (0.979, 1.824)	1.228 (0.896, 1.682)
* P* for trend		<.0001	.0100	.0449
Hypertension				
Serum ferritin/(10 μg/L)		1.031 (1.027, 1.035)*	1.014 (1.009, 1.019)*	1.012 (1.007, 1.016)*
Q1 (≤36 μg/L)	905 (22.09)	1 (ref)	1 (ref)	1 (ref)
Q2 (36.1–74 μg/L)	1164 (28.85)	1.298 (1.135, 1.483)*	1.112 (0.967, 1.280)	1.089 (0.950, 1.248)
Q3 (74.1–147 μg/L)	1605 (40.37)	1.944 (1.706, 2.216)*	1.330 (1.135, 1.559)*	1.283 (1.099, 1.499)*
Q4 (>147 μg/L)	1915 (47.66)	2.713 (2.380, 3.092)*	1.531 (1.282, 1.828)*	1.424 (1.197, 1.693)*
* P* for trend		<.0001	<.0001	<.0001

Case displays the number of participants with High SBP/DBP or hypertension and percentage (%) in parenthesis. Serum ferritin/(10 μg/L) represents dividing the continuous variable ferritin by 10 to observe the change of the dependent variable with each increase of 10 μg/L of the serum ferritin.

Model 1 did not adjust any confounders.

Model 2 adjusted demographic characteristics including age, gender, race, family income, education level, BMI, smoking status, physical activity and health insurance status.

Model 3 further adjusted total cholesterol, glycohemoglobin, albumin, white blood cell count, liver disease, kidney disease and dyslipidemia.

* *P* < .05.

### 3.4. Subgroup analysis

**Figure 3.**
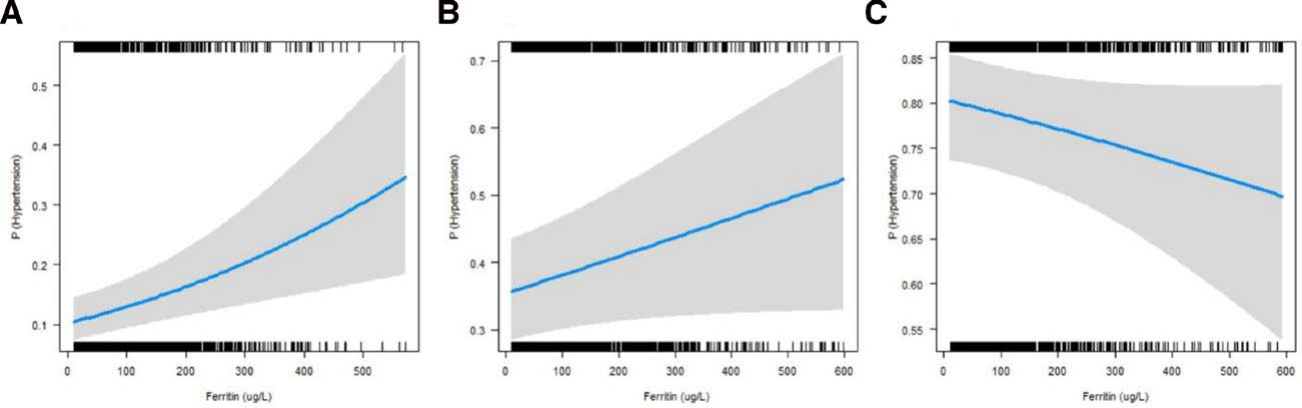
Association between serum ferritin (μg/L) and hypertension in subgroup analysis by age. A: Association between serum ferritin and hypertension in participants aged between 20 and 40; B: Association between serum ferritin and hypertension in participants aged between 40 and 60; C: Association between serum ferritin and hypertension in participants aged over 60; All models adjusted gender, race, family income, education level, BMI, smoking status, physical activity, health insurance status, total cholesterol, glycohemoglobin, albumin, white blood cell count, liver disease, kidney disease and dyslipidemia.

In different age and gender subgroups, stratified analysis revealed differences in the association between serum ferritin levels and high SBP, high DBP, and hypertension (Table 4). Serum ferritin levels in the males did not significantly associate with neither high SBP, high DBP, nor hypertension, whereas they showed statistically significant association in females. The risk of hypertension was 1.229 times higher in the Q3 (95% CI: 1.032–1.462) and 1.427 times higher in the Q4 (95% CI: 1.099–1.853) compared to the Q1 in females, and the risk of hypertension increased with ascending serum ferritin levels, with a statistically significant trend (*P* for trend = 0.0042). In all age groups, serum ferritin levels were significantly associated with hypertension (Fig. 3A and B). Specially, High serum ferritin levels were protective factors against hypertension in people over 60 (Fig. 3C); in this group comparison to the Q1, the Q4 had a 38.3% lower risk of hypertension (OR: 0.617, 95% CI: 0.463–0.822), but high serum ferritin levels significantly elevated the risk of hypertension in other age groups (Table 4).

**Table 4:** Association between serum ferritin levels and risk of High SBP, High DBP and Hypertension stratified by age and gender.

	High SBP		High DBP		Hypertension	
	Case	OR (95% CI)	Case	OR (95% CI)	Case	OR (95% CI)
Stratified by age						
20–40						
Serum ferritin/(10 μg/L)		1.030 (1.013, 1.047)*		1.026 (1.006, 1.047)*		1.016 (1.005, 1.028)*
Q1 (≤36 μg/L)	42 (1.64)	1 (ref)	50 (1.96)	1 (ref)	258 (10.09)	1 (ref)
Q2 (36.1–74 μg/L)	30 (1.45)	0.684 (0.427, 1.095)	35 (1.69)	0.755 (0.462, 1.233)	215 (10.37)	1.123 (0.904, 1.395)
Q3 (74.1–147 μg/L)	43 (2.88)	1.015 (0.565, 1.825)	51 (3.42)	1.234 (0.762, 1.998)	224 (15.01)	1.529 (1.182, 1.978)*
Q4 (>147 μg/L)	61 (5.90)	1.272 (0.684, 2.365)	77 (7.45)	1.416 (0.810, 2.476)	221 (21.37)	1.775 (1.270, 2.482)*
* P* for trend		0.1388		0.1683		0.0023
41–60						
Serum ferritin/(10 μg/L)		1.010 (1.001, 1.019)*		1.007 (0.996, 1.017)		1.012 (1.006, 1.019)*
Q1 (≤36 μg/L)	121 (10.72)	1 (ref)	68 (6.02)	1 (ref)	337 (29.85)	1 (ref)
Q2 (36.1–74 μg/L)	141 (12.25)	1.075 (0.774, 1.492)	84 (7.30)	0.948 (0.598, 1.502)	385 (33.45)	1.092 (0.852, 1.400)
Q3 (74.1–147 μg/L)	210 (16.06)	1.322 (0.959, 1.821)	147 (11.24)	1.177 (0.835, 1.660)	525 (40.14)	1.225 (0.951, 1.579)
Q4 (>147 μg/L)	240 (16.52)	1.439 (0.988, 2.096)	168 (11.56)	1.141 (0.768, 1.694)	620 (42.67)	1.438 (1.097, 1.886)*
* * *P* for trend		.0504		.4677		.0076
≥60						
Serum ferritin/(10 μg/L)		0.998 (0.992, 1.005)		0.991 (0.979, 1.003)		0.999 (0.994, 1.005)
Q1 (≤36 μg/L)	188 (45.85)	1 (ref)	21 (5.12)	1 (ref)	310 (75.61)	1 (ref)
Q2 (36.1–74 μg/L)	360 (44.39)	0.982 (0.734, 1.312)	42 (5.18)	0.548 (0.309, 0.972)*	564 (69.54)	0.730 (0.526, 1.012)
Q3 (74.1–147 μg/L)	523 (44.47)	0.942 (0.670, 1.325)	67 (5.70)	0.645 (0.324, 1.283)	856 (72.79)	0.703 (0.493, 1.004)
Q4 (>147μg/L)	630 (41.15)	0.946 (0.696, 1.285)	105 (6.86)	0.687 (0.334, 1.411)	1074 (70.15)	0.617 (0.463, 0.822)*
* * *P* for trend		.7242		.9049		.0048
* P* for effect modification		<.0001		.0015		<.0001
Stratified by gender						
Male						
Serum ferritin/(10 μg/L)		1.005 (0.997, 1.012)		1.012 (1.002, 1.022)*		1.006 (1.000, 1.012)
Q1 (≤36 μg/L)	72 (25.35)	1 (ref)	18 (6.34)	1 (ref)	148 (52.11)	1 (ref)
Q2 (36.1–74 μg/L)	172 (21.99)	1.138 (0.732, 1.769)	47 (6.01)	0.936 (0.405, 2.163)	322 (41.18)	0.865 (0.564, 1.327)
Q3 (74.1–147 μg/L)	325 (19.36)	1.255 (0.780, 2.017)	139 (8.28)	1.266 (0.579, 2.768)	679 (40.44)	0.941 (0.631, 1.402)
Q4 (>147 μg/L)	536 (19.71)	1.206 (0.752, 1.932)	276 (10.15)	1.547 (0.677, 3.536)	1197 (44.02)	0.981 (0.638, 1.508)
* P* for trend		.7224		.0305		.5458
Female						
* * Serum ferritin/(10 μg/L)		1.009 (1.001, 1.017)*		0.999 (0.984, 1.014)		1.019 (1.010, 1.027)*
* * Q1 (≤36 μg/L)	279 (7.32)	1 (ref)	121 (3.17)	1 (ref)	757 (19.86)	1 (ref)
* * Q2 (36.1–74 μg/L)	359 (11.04)	0.967 (0.759, 1.234)	114 (3.50)	0.831 (0.596, 1.159)	842 (25.88)	1.082 (0.925, 1.266)
* * Q3 (74.1–147 μg/L)	451 (19.63)	1.041 (0.818, 1.324)	126 (5.49)	1.111 (0.800, 1.543)	926 (40.31)	1.229 (1.032, 1.462)*
* * Q4 (>147 μg/L)	395 (30.41)	1.260 (0.966, 1.643)	74 (5.70)	0.770 (0.504, 1.174)	718 (55.27)	1.427 (1.099, 1.853)*
* P* for trend		.0399		.4380		.0042
* P* for effect modification		.3837		.3240		.4893

Case displays the number of participants with High SBP/DBP or hypertension and percentage (%) in parenthesis. Serum ferritin/ (10 μg/L) represents dividing the continuous variable ferritin by 10 to observe the change of the dependent variable with each increase of 10 μg/L of the serum ferritin.

Weighted logistic regression model adjusted age, gender, race, family income, education level, BMI, smoking status, physical activity, health insurance status, total cholesterol, glycohemoglobin, albumin, white blood cell count, liver disease, kidney disease and dyslipidemia.

Testing for linear trend is performed through weighted logistic model by considering the serum ferritin quartiles as a continuous variable.

Testing for the effect modification is performed through likelihood ratio test.

* *P* < .05.

In participants with abnormal liver function, the association between serum ferritin levels and hypertension was not statistically significant, with or without adjustment for covariates. However, each 10 μg/L increase in serum ferritin was positively associated with a 2.1% increase in the risk of hypertension (OR: 1.021, 95% CI: 1.009–1.033) in individuals with abnormal liver function when ferritin was included in model as continuous variable after adjusting covariates. In subjects with renal disease and dyslipidemia, the association between serum ferritin levels and hypertension was not significantly different from that in the general population, and high levels of serum ferritin concentrations significantly increased the risk of developing hypertension (Table 5).

**Table 5:** Subgroup analysis of association between serum ferritin levels and risk of Hypertension.

	Case	Model 1	Model 2	Model 3
Participants with abnormal liver function				
Serum ferritin/(10 μg/L)		1.015 (1.004, 1.027)*	1.020 (1.008, 1.033)*	1.021 (1.009, 1.033)*
Q1 (≤36 μg/L)	43 (43.88)	1 (ref)	1 (ref)	1 (ref)
Q2 (36.1–74 μg/L)	64 (42.38)	0.643 (0.308, 1.342)	0.620 (0.294, 1.307)	0.572 (0.274, 1.193)
Q3 (74.1–147 μg/L)	93 (47.69)	0.750 (0.404, 1.392)	0.817 (0.413, 1.616)	0.799 (0.393, 1.623)
Q4 (>147 μg/L)	183 (52.44)	1.221 (0.636, 2.345)	1.452 (0.685, 3.079)	1.423 (0.659, 3.072)
* P* for trend		.0356	.0177	.0015
* P* for effect modification		.0304	.1331	.0874
Participants with chronic kidney disease				
Serum ferritin/(10 μg/L)		1.027 (1.019, 1.034)*	1.013 (1.005, 1.022)*	1.011 (1.003, 1.019)*
Q1 (≤36 μg/L)	275 (25.42)	1 (ref)	1 (ref)	1 (ref)
Q2 (36.1–74 μg/L)	363 (32.70)	1.455 (1.153, 1.835)*	1.199 (0.933, 1.540)	1.118 (0.881, 1.418)
Q3 (74.1–147 μg/L)	543 (43.72)	2.044 (1.624, 2.572)*	1.354 (1.034, 1.773)*	1.263 (0.971, 1.643)
Q4 (>147 μg/L)	684 (50.11)	2.582 (2.091, 3.188)*	1.490 (1.134, 1.959)*	1.310 (1.001, 1.713)*
* P* for trend		<.0001	.0124	.0022
* P* for effect modification		.4211	.8052	.8929
Participants with dyslipidemia				
Serum ferritin/(10 μg/L)		1.040 (1.034, 1.047)*	1.017 (1.009, 1.024)*	1.014 (1.007, 1.020)*
Q1 (≤36 μg/L)	500 (18.38)	1 (ref)	1 (ref)	1 (ref)
Q2 (36.1–74 μg/L)	598 (23.67)	1.252 (1.051, 1.492)*	1.102 (0.928, 1.310)	1.064 (0.898, 1.260)
Q3 (74.1–147 μg/L)	794 (36.81)	2.082 (1.774, 2.443)*	1.290 (1.073, 1.550)*	1.230 (1.020, 1.484)*
Q4 (>147 μg/L)	913 (48.62)	3.309 (2.720, 4.026)*	1.471 (1.170, 1.851)*	1.359 (1.083, 1.706)*
* P* for trend		<.0001	.0015	.0023
* P* for effect modification		<.0001	.2988	.2839

Case displays the number of participants with High SBP/DBP or hypertension and percentage (%) in parenthesis. Serum ferritin/ (10 μg/L) represents dividing the continuous variable ferritin by 10 to observe the change of the dependent variable with each increase of 10 μg/L of the serum ferritin.

Model 1 did not adjust any confounders; Model 2 adjusted demographic characteristics including age, gender, race, family income, education level, BMI, smoking status, physical activity and health insurance status; Model 3 further adjusted total cholesterol, glycohemoglobin, albumin, white blood cell count in common. Participants with chronic liver disease further adjusted kidney disease and dyslipidemia. Participants with chronic kidney disease further adjusted liver disease and dyslipidemia. Participants with dyslipidemia further adjusted kidney disease and liver disease.

Testing for linear trend is performed through weighted logistic model by considering the serum ferritin quartiles as a continuous variable. Testing for the effect modification is performed through likelihood ratio test.

* *P* < .05.

## 4. Discussion

Iron plays an important role in maintaining physiological homeostasis in the body, and influenced by multiple factors; however, excess iron can produce free radical to damage tissue.^[31]^ Ferritin, one of the key proteins regulating iron homeostasis, is a widely available clinical biomarker to evaluate iron status.^[32,33]^ Several studies have reported an association between serum ferritin concentration and hypertension.^[34–38]^ Although there has been some researches on the relationship between serum ferritin and hypertension, it is still confusion. To determine the relationship of serum ferritin level and hypertension, weighted regression analysis and more population were conducted. Our study supported that high serum ferritin levels are closely related to hypertension.

In this cross-sectional study conducted in hypertensive patients, we suggested a close relationship between serum ferritin and hypertension, more pronounced in females with high serum ferritin. The levels of serum ferritin in hypertension and high SBP and high DBP groups were significantly higher than those in non-hypertension and normal SBP and normal DBP (Table 2). In addition, when serum ferritin is a continuous variable in 3 models, this association remained significant, even if adjusted the common factors between hypertension and serum ferritin, including age, gender, race, family income, education level, BMI, smoking status, physical activity, health insurance status, total cholesterol, glycohemoglobin, albumin, white blood cell count, liver disease, kidney disease and dyslipidemia (Table 3). Moreover, our results indicated that the proportion of subjects with elevated serum ferritin increases with age; With rising serum ferritin levels, SBP, DBP, and hypertension prevalence all trended significantly upward, and after adjusting for covariates, serum ferritin levels remained to be closely associated with high SBP and hypertension but not with high DBP (Table 4). Previously, most researches from different countries had similar conclusions on the associations of serum ferritin and hypertension,^[34–36]^ in the study of Kim et al,^[35]^ they found that serum ferritin was a significant predictor of hypertension in middle-aged men and this association was significant even after adjustment for age, BMI and baseline blood pressure. However, this trend does not included those aged 60 years and older in our study. Specially, as is shown in Figure 3C, the serum ferritin level is negatively correlated with the incidence of hypertension in people aged 60 years and more, which was 38.3% lower risk of hypertension, this means that, high levels of serum ferritin may be a protective factor for these patients of hypertension. This condition is uncommon and inconsistent with previous studies. A previous retrospective cross-sectional study indicated that ferritin concentrations was positively associated with blood pressure among the elderly in South Africa.^[37]^ Another prospective cohort study found that serum ferritin and serum soluble transferrin receptor concentration had a non-significant effect on incident hypertension.^[38]^ Two cross-sectional studies and one cohort study in Korea all suggested that serum ferritin was positively associated with the prevalence of hypertension.^[35,36,39]^ This inconsistency may be due to a number of factors, including the heterogeneity of the study populations and the study regions, as well as the research methods.

One of the possible mechanisms about the association between serum ferritin levels and hypertension maybe is that inflammation and oxidative stress links serum ferritin together with hypertension. We assume that the interaction is biologically plausible. Firstly, inflammation, oxidative stress and immunity all play the important roles in the hypertension.^[18,40–42]^ Furthermore, serum ferritin concentrations reflect not only body iron stores but also inflammation, and serve as a catalyst and amplifier of the inflammatory response.^[43]^ High serum ferritin via Fenton reactions to produce hydroxyl free radical, and lead to oxidative damage and consequent further cellular damage, which leads to inflammation, vascular endothelial damage and consequently atherosclerosis, leading to the change of vascular elasticity and inner diameter, and then risk of hypertension can be increased.^[15,44–46]^ Moreover, they all have a very close relationship with diet and environment. These evidences supported the correlation between serum ferritin and hypertension.

In addition, a study indicated that serum ferritin was positively correlated to DBP in adult women,^[47]^ but in our study, serum ferritin levels in females showed statistically associate with high SBP and hypertension, whereas had no association with DBP. Therefore, more researches are needed on the relationship between serum ferritin and DBP and SBP.

Metabolic syndrome is a very complex disease state, and there are many studies have shown that moderately elevated serum ferritin levels are associated with an increased prevalence of metabolic syndrome or some of its components, such as high blood pressure.^[48–52]^ In our study, we also found that total cholesterol, HDL, LDL, triglycerides, gyrated hemoglobin and albumin also showed an ascending trend with increasing serum ferritin levels.

The major limitation of our study still exists. Despite the support of cohort studies, causal inferences of serum ferritin with hypertension remain limited. We can’t observe the dynamic change of serum ferritin content in the course of hypertension and the causal relationship between serum ferritin and the change of disease condition by retrospective analysis. In addition, although we have taken into account many possible confounding factors, we cannot fully consider all potential confounding factors due to the limited variables in the database as well as the retrospective nature of the data. Many diseases have an impact on serum ferritin concentrations, such as inflammation, tumors and metabolic syndrome,^[53,54]^ but in our study, we did not exclude these patients or adjusted these factors. Moreover, the occurrence and development of hypertension is a very complex pathophysiological process, which is related to genes, environment, diet, lifestyle, family history and other factors. However, in this study, we only analyzed the relationship between serum ferritin and hypertension. Therefore, it is necessary to carry out more clinical and laboratory studies to investigate the association between serum ferritin and blood pressure as well as the exact mechanism.

## 5. Conclusions

This study further confirmed the relationship between serum ferritin and hypertension in American adults. Continuous elevated serum ferritin levels were closely related to hypertension. Large sample prospective studies and more basic mechanistic researches are warranted to further elucidate the potential mechanisms underlying the associations between serum ferritin and hypertension in the future.

## Acknowledgments

We want to express our gratitude to all the participants, primary care doctors and nurses who took part in the survey.

## Author contributions

**Conceptualization:** Junting Liu.

**Data curation:** Tao Li, Dongqing Hou.

**Formal analysis:** Feilong Chen, Guimin Huang.

**Funding acquisition:** Shaoli Li, Junting Liu.

**Methodology:** Yijing Cheng, Wenqian Liu.

**Supervision:** Wenqian Liu.

**Validation:** Tao Li, Yijing Cheng, Dongqing Hou.

**Writing – original draft:** Shaoli Li, Feilong Chen.

**Writing – review & editing:** Tao Xu.
